# Predictors of male loneliness across life stages: an Australian study of longitudinal data

**DOI:** 10.1186/s12889-024-18770-w

**Published:** 2024-05-10

**Authors:** Ferdi Botha, Marlee Bower

**Affiliations:** 1 Melbourne Institute: Applied Economic & Social Research, The University of Melbourne, Melbourne, Australia; 2 ARC Centre of Excellence for Children and Families Over the Life Course, Queensland, Australia; 3 The Matilda Centre for Research in Mental Health and Substance Use, The University of Sydney, Camperdown, Australia

**Keywords:** Loneliness, Males, Social isolation, Life course, Longitudinal data, Australia

## Abstract

**Background:**

Despite growing recognition of loneliness as a global public health concern, research on its occurrence and precipitants among men across different life stages remains limited and inconclusive. This study aims to address this gap by investigating the prevalence and predictors of loneliness among a large, representative data set of Australian adult men.

**Methods:**

The study used longitudinal data from waves 2–21 of the Household, Income and Labour Dynamics in Australia (HILDA) Survey, including men aged 15–98. Estimating linear fixed effects regressions that account for unobserved time-invariant individual heterogeneity, a single-item measure of loneliness was regressed on a set of selected explanatory variables over different parts of the life course.

**Results:**

Increased social isolation, romantic partnership dissolution, having a long-term disability, and stronger beliefs that the man, rather than the woman, should be the breadwinner of the household, are associated with greater loneliness. Frequent social connection, having a romantic partner, and high neighbourhood satisfaction are protective against loneliness. The findings also reveal several differences in the predictors of loneliness over the life course. Job security is especially important for younger men, whereas for older men volunteering and less conservative gender role attitudes are important factors that can decrease loneliness.

**Conclusions:**

The results emphasise the need to consider age-specific factors and societal expectations in understanding and addressing loneliness amongst men. Additionally, the findings underscore the importance of raising awareness about the impact of societal norms and expectations on men's mental health. The results offer valuable insights for policymakers, healthcare providers, and researchers to develop effective strategies and support systems to combat loneliness and promote well-being among men.

**Supplementary Information:**

The online version contains supplementary material available at 10.1186/s12889-024-18770-w.

## Background

Loneliness is an aversive emotional response related to a perceived discrepancy between the social connections one has and those they desire [1]. Loneliness is distinct from objective social isolation: a person can feel lonely despite having extensive social networks, and similarly, can have very few social connections without experiencing loneliness. Mostly, loneliness is a short-lived phenomenon, and a normal response to social isolation, spurring efforts to re-establish meaningful connection [2]. However, prolonged periods of loneliness can be associated with serious physical health conditions like cardiac disease and immune deficiency and mental health conditions like anxiety, depression and even suicide [3, 4].

Despite increasing recognition and knowledge of loneliness as a global public health concern [5], there remains a notable relative lack of research investigating its manifestation amongst men. This is notwithstanding the fact that some research suggests men may experience similar or even higher rates of loneliness relative to women [6, 7]. Men are often less likely than women to openly admit to feeling lonely, making it harder for them to seek necessary help and support [8]. Given the public health implications of loneliness, it is imperative to gain a deeper understanding of the underlying reasons for loneliness in men to tailor effective prevention and age- and gender-appropriate interventions, policies and strategies.

### Patterns of male loneliness

Existing research suggests that the likelihood of men experiencing loneliness is likely to differ according to their age [9]. While some research suggests that male loneliness worsens linearly with age [10, 11], one study including a large, albeit non-representative, sample of 46,054 participants in 237 countries found loneliness tended to improve with age [7]. Other studies have identified unique patterns. For instance, research using a Norwegian longitudinal sample reported that men experience peaks of loneliness during mid-life (around age 40) and later in old age (around 80) [12]. Additionally, some research has identified a non-linear, shallow ‘U-shaped’ trend, with young and elderly adults tending to experience greater loneliness than those in middle age [13, 14]. Interestingly, the opposite phenomenon has been found in a non-western sample of Chinese men, where a reverse ‘U-shape’ showed a peak of loneliness at age 55 [15]. The variation between these findings underscores the influence of social and potentially cultural context on the causes of loneliness in men across different age brackets. There is currently scant research examining the predictors of male loneliness at different life stages across high-quality, representative datasets [9, 16].

### Predictors of male loneliness

Research has consistently identified the role of objective social isolation in increasing male loneliness. In terms of friendship, having fewer friends, lacking close male friendships and having limited opportunities for social interactions have all been associated with increased loneliness [17–23]. While much of this research has focused on younger men, we may anticipate that poor-quality or lost friendships may also foster loneliness amongst older men. In terms of intimate relationships, the absence of a romantic partner, undergoing divorce, or the death of a partner have been linked to higher levels of loneliness among adult men [10, 17, 18, 24–27].

Men’s living arrangements and living locations have also been identified as a potential predictor of loneliness. One study amongst youth found that the region in which a person resides, rather than socio-economic status or level of urban-ness, plays a pivotal role in determining their experience of loneliness, a finding which the authors argued underscored the importance of a sense of belonging to a place as a protective factor against loneliness [19]. Therefore, living in a neighbourhood you feel you belong is likely to buffer against loneliness, whereas living in a place you do not feel you belong may exacerbate loneliness [28]. While living alone tends to be correlated with increased loneliness [28], this association can be dependent on various other factors like income and marital status, which can mitigate the impact of living alone by providing other opportunities for meaningful social interaction [9]. Finally, male single parents tend to be at high likelihood of loneliness, relating to men in other household arrangements [13]. 

Work-related factors, including insecure employment through temporary contracts and remote work, have also been associated with loneliness [29, 30]. Recent research has also shown that the relationship between loneliness and employment can be bidirectional: being unemployed can lead to loneliness and isolation via increasing feelings of exclusion from the employed and stigma-related shame [31]. Conversely, people experiencing sustained loneliness are more likely to be unemployed at subsequent follow-ups [32]. The transition out of the workforce (e.g., through retirement) can disrupt social relationships and threaten established social identities, further contributing to loneliness [33]. However, building a sense of purpose has been linked with reduced loneliness amongst men of retirement age [14], which can be achieved through loneliness-reducing activities like volunteering [34–36].

Finally, there is also some evidence that the pressure for men to fit into mainstream cultural and social norms may drive loneliness. For example, men who belong to marginalised and stigmatised social categories, such as minority cultural backgrounds or non-heterosexual orientations, tend to experience increased loneliness [19]. Additionally, research indicates that mainstream societal expectations around masculine gender norms can discourage men from acknowledging their loneliness or seeking help when they do experience it [8, 23, 26, 37–40].

Many of the predictors described above may differ across men of different age brackets. For example, amongst social isolation predictors it can be theorised that the absence of certain kinds of social connections would have age-specific implications for loneliness. Factors like the death of a partner may disproportionately contribute to loneliness in older age (e.g., 65 +), while societal expectations regarding partnership and parenthood may heighten feelings of loneliness during middle age [9]. Concerning predictors related to cultural and masculinity norms, certain beliefs, such as a belief in the importance of men being primary ‘breadwinners’, may be especially pertinent to middle-aged men, whereas the social value of stable employment may be less critical for retirement-aged men than their younger counterparts. However, it is unclear whether other social norms around age are associated with loneliness; research data from the US and Germany have yielded mixed evidence regarding the ‘age-normative’ theory of loneliness, which posits that individuals who deviate from age-specific social and cultural norms may experience increased loneliness [9, 16].

### The present research

In this context, our research aims to address the following critical question: What are patterns and predictors of loneliness amongst Australian men of different age groups?

## Data and methods

### Data

We used data from waves 2–21 of the Household, Income and Labour Dynamics in Australia (HILDA) Survey, spanning the years 2002 to 2021. The HILDA Survey is a large annual national longitudinal survey initiated in 2001 and currently follows about 7,500 households in which all members aged 15 and over are interviewed [41]. We began with the second wave because some key explanatory variables, notably related to certain major life events, are only available since this wave. Because our estimation method only estimates changes in the outcome variable for given changes in the independent variables, we only keep individuals observed for at least two periods. The final analytical sample comprises a total of 118,667 person-year observations for 12,117 unique individuals.

### Outcome variables

Given evidence about the stigma of reporting loneliness among males [39], we used two loneliness measures that form part of the self-completion questionnaire (SCQ) in the HILDA Survey. The first variable was a single-item direct measure based on respondents’ level of agreement with the statement: “I often feel very lonely” (also see [42]) rated on a scale ranging from 1 (completely disagree) to 7 (completely agree), with higher scores consistent with greater reported loneliness.

The second variable is a multi-item measure of loneliness, developed by Manera et al. [43], consisting of three items that comprise direct and indirect measures of loneliness, and all are measured on a Likert scale ranging from 1 (completely disagree) to 7 (completely agree). The statements are: (i) “People don’t come to visit me as often as I would like”; (ii) “I often need help from other people but can’t get it”; and (iii) “I often feel very lonely,” which together reflect loneliness due to unmet social needs [43]. Factor analysis revealed that all three items load on the same underlying factor. In this sample, the Cronbach alpha for this set of items was moderate at 0.66. We constructed the multi-item loneliness variable using the average response to the three statements so that the variable still ranged from 1 to 7 and is increasing in the degree of reported loneliness.

**Fig. 1:**
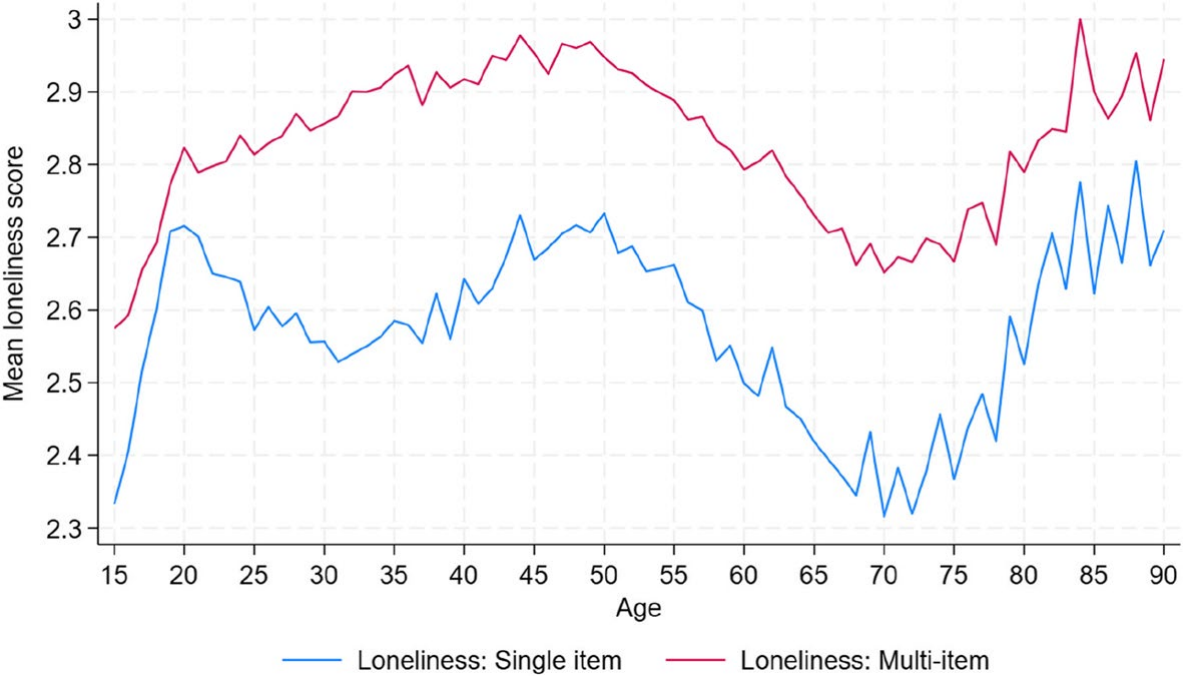
Mean loneliness score, by age. Note: HILDA Survey, waves 2–21, males aged 15–90. The figure displays the mean loneliness score, ranging from 1 (low)-7(high), for each measure

In Fig. 1 below we report average loneliness across age for both loneliness measures. However, because the regression results are broadly similar for the single-item and multi-item measure with similar conclusions, we only report and discuss the results of the single-item loneliness measure as outcome. Full regression results for the multi-item loneliness outcome are available in Tables S1 and S2 in the Supplementary Information.

### Explanatory variables

The explanatory variables were selected based on previous literature exploring the possible determinants of loneliness amongst males. The main *demographic characteristic* of interest is the respondent’s age. For the purposes of the analysis in this paper, age groups were split into 15–24, 25–34, 35–44, 45–54, 55–64, and 65 and older; an assumption was made that each of these groups represented a reasonable coverage of each life stage.

We included factors related to *employment and income characteristics*, which consisted of labour market status, job security, and household income. Labour market status consisted of indicators for whether a respondent is employed, unemployed, and not in the labour force. Perceived job security was elicited from the statement: “I have a secure future in my job.” Responses ranged from 1 (completely disagree) to 7 (completely agree). Household income denotes real disposable equivalized annual household income that adjusts for household composition.

In relation to *social life characteristics*, we included indicators for whether a respondent was partnered, whether a person volunteered their time to charity or helping others, whether a person was a member of a club or hobby association, had experienced a major life event related to the death of a close friend or separation from a spouse or partner in the past year, the quantity of friends, and frequency of social connection [43]. Quantity of friends was measured using respondents’ self-reported perceptions of the extent to which they agreed with the statement: “I seem to have many friends.” Responses ranged from 1 (completely disagree to 7 (completely agree). Frequency of social connection was based on the question: “In general, about how often do you get together socially with friends or relatives not living with you?” We grouped response categories into “at least once per week,” “at least once per month,” and “once every three months or longer.”

Indicators related to *living arrangements and location* included household type, region of residence, and neighbourhood satisfaction. Household type refers to household composition and includes “couples without children,” “couples with children,” single-parent households,” and “other household types” (which includes “other related family without children,” “lone person,” “group households,” and “multi-family households”). Region of residence denoted whether a person resided in a rural, semi-urban, or major urban area. Neighbourhood satisfaction was derived from an item that asks respondents to state how satisfied they were with the neighbourhood in which they live. Responses ranged from 0 (totally dissatisfied) to 10 (totally satisfied).

Finally, among other *personal factors* we included an indicator for whether respondents reported having a long-term disability, and the degree to which they held stereotypical masculinity beliefs. The latter was obtained from people’s level of agreement with the statement: “It is not good for a relationship if the woman earns more than the man.” Responses ranged from 1 (strongly disagree) to 7 (strongly agree), where we understood a higher level of agreement implying stronger stereotypical masculinity beliefs around gender roles. The latter item was asked in waves 5, 8, 11, 15, and 19. The following section includes a brief description of how observations in the alternative waves were dealt with.

## Methods

The longitudinal nature of the data was exploited by estimating linear fixed effects models that controlled for unobserved time-invariant individual heterogeneity. For ease of interpretation, all models were estimated via OLS. The loneliness measure was then regressed on the selected explanatory variables, controlling for individual fixed effects. We therefore exploit the variation originating from within-person *changes* in the explanatory variables and how those changes are associated with changes in loneliness.

In addition to the main explanatory variables, all models additionally included year dummies to control for period effects. Furthermore, during much of the fieldwork in 2020 and 2021, some states and territories were in lockdown due to the COVID-19 pandemic. In 2020, this was the state of Victoria. In 2021, the lockdowns were in Victoria, New South Wales, and the Australian Capital Territory. We therefore added a dummy indicator for whether a respondent resided in the relevant areas during 2020 and 2021.

The measure of job security was observed only for those who were employed, resulting in a relatively high number of missing observations in relation to other observed variables. To use as much information as possible for the regression analyses, we included a category for whether the job security response was missing, and additionally included a dummy to control for missing job security. Similarly, questions on masculinity norms were not asked in all survey waves. Where possible, we replaced a missing observation with information from the nearest available wave and include a dummy to control for missing information on masculinity norms.

The full model specification is:

1 $$y_{it}{=\beta X}_{it}+V_i+\varepsilon_{it}$$

where *y*_*it*_ is reported loneliness for person *i* at time *t*, ***X***_***it***_ is a vector of explanatory variables and indicators, *ν*_*i*_ is the individual fixed effect, and *ε*_*it*_ is the error term.

We first estimated a general model for the entire male sample that controlled for age, with the purpose of gaining insights into the determinants of male loneliness net of age. Following that, we ran linear fixed effects regressions for each age group separately to determine whether the predictors of loneliness depended on a person’s life stage. Finally, we also estimated fixed effects logit models for a binary loneliness response. The conclusions are generally similar to our main results, and an explanation of the binary outcome construction as well as the full results of these models are available in the Supplementary Information (Tables S3 and S4).

## Results

### Summary statistics

Summary statistics were reported in Table 1. The mean loneliness score was slightly higher for the multi-item measure (2.86) compared to the single item measure (2.63). In terms of some other selected statistics, the average age was approximately 44, just under three-quarters of the sample were employed, 63 percent were partnered, and 39 percent were a member of a club or hobby association.

**Table 1:** Summary statistics

	**Mean**	**Std. Dev**	**Min**	**Max**
Loneliness: Single-item	2.63	1.72	1	7
Loneliness: Multi-item	2.86	1.30	1	7
Age	44.24	18.15	15	98
Unemployed	0.04	0.18	0	1
Employed	0.70	0.46	0	1
Not in labour force	0.27	0.44	0	1
Job security	4.91	1.63	1	7
Log household income	1.84	0.47	0	4.68
Partnered	0.63	0.48	0	1
Volunteer	0.16	0.36	0	1
Member of club	0.39	0.49	0	1
Life event: death of close friend	0.10	0.30	0	1
Life event: separated from spouse or partner	0.03	0.18	0	1
I seem to have a lot of friends	4.39	1.66	1	7
Frequency of social connection (%)				
Once every 3 months or longer	13.19			
At least once a month	32.00			
At least once a week	54.81			
Household type (%)				
Couple without children	26.85			
Couple with children	47.60			
Single parent	7.56			
Other household type	17.99			
Major urban	0.72	0.45	0	1
Other urban	0.18	0.39	0	1
Rural	0.28	0.45	0	1
Satisfaction with neighbourhood	7.85	1.65	0	10
Long-term disability	0.16	0.37	0	1
Stereotypical masculinity beliefs	2.51	1.60	1	7

Weighted (using SCQ respondent population weights) data from the HILDA Survey, Waves 2–21, males aged 15–98. *N* = 118,667, except for job security (*N* = 81,197) and masculinity beliefs (*N* = 113,942). See Methods section for an explanation of how these two variables are included in the regression analyses

Figure 1 plots the average loneliness scores of the two loneliness measures by age. Average levels of loneliness were clearly higher for the multi-item measure at all ages, which is consistent with evidence that men tend to underreport loneliness when asked about it directly [39], as in the single item measure. In general, mean loneliness peaks at around middle age, declines from age 50, after which loneliness starts rising again from age 70.

### Regression analysis

Table 2 reports the fixed effects regression results for the determinants of loneliness in the general male sample, controlling only for age in column [1] and adding the remaining set of explanatory variables in column [2]. In column [1], loneliness tended to be significantly lower among 15–24-year-olds as compared to those aged 25–54 by between 0.07- and 0.17 points on the 1–7 scale. The age differences in loneliness for the 25–44 groups remained once covariates were added in column [2], and loneliness was on average 0.23 points lower among males aged 65 and older relative to those aged 15–24.

**Table 2:** Determinants of loneliness, all age groups

	**(1)**	**(2)**
Age group (ref: 15–24)		
25–34	0.072*** (0.026)	0.141*** (0.027)
35–44	0.145*** (0.035)	0.127*** (0.039)
45–54	0.170*** (0.042)	0.066 (0.049)
55–64	0.062 (0.047)	-0.090 (0.059)
65 and older	-0.047 (0.052)	-0.234*** (0.070)
Labour force status (ref: Unemployed)		
Employed		-0.019 (0.034)
Not in labour force		-0.099*** (0.028)
Job security		-0.043*** (0.004)
Log household income		-0.003 (0.015)
Partnered		-0.518*** (0.030)
Volunteer		-0.004 (0.015)
Member of club		-0.028** (0.012)
Life event: death of close friend		0.029** (0.014)
Life event: separated from spouse or partner		0.343*** (0.029)
I seem to have many friends (1–7)		-0.133*** (0.003)
Frequency of social connection (ref: Less than once a month)		
At least once a month		-0.160*** (0.017)
At least once a week		-0.265*** (0.018)
Household type (ref: Couple without children)		
Couple with children		0.107*** (0.018)
Single parent		0.187*** (0.042)
Other household type		0.273*** (0.028)
Region of residence (ref: Rural)		
Major urban		-0.015 (0.041)
Other urban		0.025 (0.044)
Satisfaction with neighbourhood (1–10)		-0.045*** (0.004)
Long-term disability		0.125*** (0.018)
Stereotypical masculinity beliefs		0.023*** (0.005)
COVID-19 lockdown		-0.033 (0.027)
Constant		4.062*** (0.083)
Mean of dependent variable	2.629	2.629
Within R^2^	0.0010	0.0466
Observations	118,667	118,667
Individuals	12,117	12,117

The dependent variable is the response to the question “I often feel very lonely” on a 1-7 scale, with higher values meaning greater loneliness. For job security and masculinity beliefs, indicator categories are included for missing observations and dummies are additionally included (but not shown) to control for missingness in these variables. Year dummies are included (but not shown) in all models. Robust standard errors are in parentheses. *p* < 0.01***, *p* < 0.05**

Individuals were less lonely if they were not in the labour force than if they were unemployed. An increase in perceived job security was associated with a reduction in loneliness, whereas there was no significant relationship between loneliness and household income. Unsurprisingly, partnership status was strongly related to loneliness: men who entered a romantic partnership on average tended to have a loneliness score of 0.52 points lower compared to men who did not have a partner. There was little evidence that volunteering affected loneliness, though the results were suggestive that becoming a member of a club or hobby group was associated with lower loneliness scores.

Experiencing the death of a close friend was associated with a slight increase in loneliness, although experiencing a separation from a partner or spouse was strongly related to increased loneliness of about 0.34 points on the 1–7 scale. As expected, a rise in the number of friends a respondent perceives having was associated with a decline in loneliness. Loneliness was also lower among people who met with friends or family often. For example, men who saw family and friends at least once a week were on average 0.27 points less lonely than men who only saw family and friends less than once per month. Men residing in households where there was a couple without children were less lonely than those in couple households with children, single parents, and those in other household types.

There were no significant relationships between residence region and loneliness scores, but respondents were significantly less lonely the more satisfied they were with the neighbourhood in which they lived. Loneliness was about 0.13 points higher among persons with a long-term disability as compared to persons without a disability, and loneliness tended to be greater among men who held more stereotypical masculinity beliefs. There was no evidence that the pandemic lockdowns affected loneliness.

We now turn to the results of regressions estimated for each age group separately (Table 3), with the objective of examining whether the determinants of loneliness differ across the life stage. There were few differences in relation to labour force status. However, within the 45–54 age group employed persons were about 0.26 points less lonely than unemployed persons. Individuals younger than 65 who reported having greater job security also report being less lonely, with a one-point increase in perceived job security being associated with a decrease in loneliness of between 0.02 and 0.06 points. A change in household income was not associated with changes in loneliness among any age group.

**Table 3:** Determinants of loneliness, by age group

	**15–24**	**25–34**	**35–44**	**45–54**	**55–64**	**65 + **
Labour force status (ref: Unemployed)						
Employed	-0.022 (0.062)	0.139 (0.092)	-0.090 (0.097)	-0.264*** (0.096)	-0.084 (0.088)	-0.139 (0.196)
Not in labour force	-0.052 (0.044)	0.083 (0.075)	-0.053 (0.097)	-0.122 (0.090)	-0.073 (0.080)	-0.302 (0.188)
Job security	-0.042*** (0.009)	-0.044*** (0.009)	-0.056*** (0.009)	-0.038*** (0.010)	-0.021** (0.011)	-0.029 (0.018)
Log household income	0.041 (0.037)	-0.055 (0.039)	0.013 (0.042)	-0.017 (0.041)	-0.047 (0.035)	-0.025 (0.036)
Partnered	-0.579*** (0.069)	-0.539*** (0.059)	-0.638*** (0.107)	-0.591*** (0.107)	-0.318** (0.135)	-0.806*** (0.143)
Volunteer	-0.009 (0.042)	0.027 (0.038)	0.027 (0.036)	0.031 (0.036)	-0.037 (0.037)	-0.119*** (0.037)
Member of club	-0.040 (0.029)	-0.033 (0.027)	-0.016 (0.030)	-0.020 (0.036)	-0.042 (0.033)	-0.017 (0.037)
Life event: death of close friend	0.085* (0.046)	-0.008 (0.046)	-0.018 (0.040)	-0.019 (0.035)	0.026 (0.031)	0.059** (0.029)
Life event: separated from spouse or partner	0.421*** (0.057)	0.344*** (0.058)	0.221*** (0.073)	0.159** (0.076)	0.200* (0.105)	0.232* (0.125)
I seem to have many friends (1–7)	-0.219*** (0.012)	-0.155*** (0.011)	-0.139*** (0.012)	-0.086*** (0.012)	-0.071*** (0.012)	-0.021* (0.011)
Frequency of social connection (ref: Less than once a month)						
At least once a month	-0.104* (0.060)	-0.286*** (0.048)	-0.119*** (0.040)	-0.146*** (0.036)	-0.104** (0.042)	-0.100** (0.042)
At least once a week	-0.231*** (0.057)	-0.366*** (0.050)	-0.241*** (0.043)	-0.244*** (0.042)	-0.173*** (0.045)	-0.132*** (0.045)
Household type (ref: Couple without children)						
Couple with children	-0.106 (0.076)	0.047 (0.037)	0.119** (0.058)	0.030 (0.056)	0.091* (0.48)	0.093 (0.075)
Single parent	-0.031 (0.093)	0.083 (0.116)	0.043 (0.147)	0.224* (0.125)	0.088 (0.146)	0.268 (0.193)
Other household type	-0.003 (0.075)	0.174*** (0.058)	0.166 (0.102)	0.146 (0.092)	0.309*** (0.091)	0.420*** (0.119)
Region of residence (ref: Rural)						
Major urban	-0.072 (0.090)	-0.085 (0.089)	0.058 (0.121)	-0.055 (0.131)	-0.134 (0.129)	0.125 (0.123)
Other urban	0.072(0.097)	-0.059 (0.098)	0.114 (0.117)	-0.026 (0.147)	-0.108 (0.137)	-0.003 (0.126)
Satisfaction with neighbourhood (1–10)	-0.039*** (0.008)	-0.030*** (0.008)	-0.045*** (0.010)	-0.054*** (0.011)	-0.031*** (0.012)	-0.026** (0.011)
Long-term disability	0.182*** (0.063)	0.241*** (0.057)	0.184*** (0.053)	0.029 (0.045)	0.090** (0.040)	0.085** (0.033)
Stereotypical masculinity beliefs	0.024 (0.017)	0.010 (0.014)	0.025* (0.014)	0.015 (0.014)	0.042*** (0.014)	-0.001 (0.011)
COVID-19 lockdown	0.079 (0.088)	-0.066 (0.066)	-0.109 (0.070)	-0.082 (0.072)	0.039 (0.071)	-0.044 (0.059)
Constant	3.989*** (0.143)	4.279*** (0.118)	4.256*** (0.229)	4.537*** (0.245)	3.823*** (0.245)	3.653*** (0.308)
Mean of dependent variable	2.629	2.674	2.672	2.709	2.582	2.485
Within R^2^	0.0844	0.0668	0.0468	0.0295	0.0188	0.0246
Observations	19,107	20,275	20,376	20,745	17,778	20,386
Individuals	4,072	4,433	4,044	3,783	3,205	2,674

The dependent variable is the response to the question “I often feel very lonely” on a 1–7 scale, with higher values meaning greater loneliness. For job security and masculinity beliefs, indicator categories are included for missing observations and dummies are additionally included (but not shown) to control for missingness in these variables. Year dummies are included (but not shown) in all models. Robust standard errors are in parentheses *p* < 0.01***, *p* < 0.05**, *p* < 0.10*

Having a romantic partner was an important determinant of loneliness among all age groups, with men who became partnered being significantly less lonely (by between 0.32 to 0.81 points) than those without a partner.

Volunteering was generally not related to loneliness, but there was evidence that volunteering decreases loneliness among the 65 and older group by 0.12 points relative to those who do not volunteer. Being a member of a club or hobby group was not related to loneliness in any age group.

Losing a close friend significantly increased loneliness on average for men aged 15–24 and 65 and older. Experiencing a separation from a partner or spouse increased loneliness among all age groups, with the effect generally larger among younger age groups.

The more men agreed with the statement that they seem to have many friends, the less lonely they were on average; this relationship is evident for men of all age categories but appeared stronger for younger age groups. Seeing non-resident friends and family more frequently led to lower loneliness scores for all age groups. For example, 15–24-year-olds who saw family and friends at least once per week were on average 0.23 points less lonely than those who only saw family and friends less than once per month.

Across most age groups, men residing in households where they were part of a couple without children tended to be less lonely than men residing in the alternative household type groups. For example, compared to couples without children, men (especially those aged 25–34 and 55 and above) living in ‘other household types’ were generally lonelier, perhaps in part because lone persons—who we may expect to report high rates of loneliness—were included under the definition of ‘other household types.’

There was no evidence of a relationship between loneliness and whether someone lived in rural or more metropolitan areas. However, the association between satisfaction with neighbourhood and loneliness was significant for all age groups, with an increase in neighbourhood satisfaction related to a decrease in loneliness. Having a long-term disability predicted loneliness in all age groups except 45–54-year-olds, as men who developed a long-term disability were lonelier than men who reported no such disability. Among the 25–34 group, for example, loneliness is about 0.24 points lower for men with a long-term disability as compared to men with no such disability. Loneliness was on average greater among people who held stronger stereotypical masculinity beliefs, though mainly among men aged 35–44 and those 65 and older. Finally, results from the pandemic lockdown indicator suggested that there was no difference in loneliness between those living in regions that experienced lockdowns and those who did not live in those regions.

## Discussion

This is the first known study to examine predictors of loneliness amongst a representative male sample across different age groups. At all adult life stages, having access to good quality and supportive social relationships, including a romantic partner and friendships and being satisfied with your local neighbourhood, were protective against loneliness. Men with a long-term disability or who had recently undergone a relationship breakdown were lonelier across life stages, with few exceptions. Other factors only impacted loneliness at specific life stages, including job security, employment, and living arrangements. The death of a close friend was associated with increased loneliness among men aged 15–24 and 65 and older, potentially due to higher mortality rates in these age groups, which affect peers left behind [44, 45]. Overall, results emphasised the need for both universal and targeted age-specific loneliness prevention and intervention strategies for male loneliness.

The study revealed varying loneliness patterns across life stages, depending on whether a single-item or multi-item loneliness measures were used. The multi-item measure consistently yielded higher average loneliness scores for men of all ages, aligning with existing findings that men often underreport loneliness. Using the single-item measure, on average loneliness exhibited peaks during young adulthood (around age 18–20) and again in middle age (late 40s). In contrast, the multi-item measure indicated that average loneliness peaked in midlife (mid-40s) and was lower during young adulthood. This discrepancy in findings may reflect generational shifts in reporting, where younger men are more willing to describe themselves as lonely despite non-direct indicators suggesting otherwise. Both measures indicated loneliness was lowest around age 70, with an increase thereafter, though not reaching the levels seen during middle age.

Our findings unveil yet another distinctive manifestation of male loneliness across various life stages. As evidenced in our introduction, loneliness rates fluctuate across life phases, contingent upon the sample. It remains imperative to gather additional evidence to ascertain whether these variations stem from measurement biases or reflect genuinely divergent culturally influenced male experiences across global regions. Further investigation is warranted to juxtapose Australian data against internationally representative datasets, shedding light on the idiosyncratic factors driving loneliness among Australian men in their late-40s. One key finding was the marked association between work and loneliness amongst men. Perceived job security was a key predictor of loneliness for men prior to retirement age, with less job security being associated with greater loneliness. This may be because men on temporary work contracts may feel less likely to invest in their workplace social context if their job circumstances are likely to change. The rise of insecure work in Australia via the ‘gig economy’ may mean that some men may need to work extra jobs to support dependents, leaving less time to socialise [46]. Additionally, being employed, rather than unemployed, was protective for loneliness amongst middle-aged men aged 45–54. This may be because of normative social expectations around men providing for families: middle-aged men (aged 35–44 and 55–64) who held beliefs supported traditional gender roles around providing for the household tended to feel lonelier amongst men who did not hold these beliefs. Amongst those who are the age most likely to have a family, the added burden of feeling solely financially responsible for family wellbeing may exacerbate loneliness.

The study also identified living arrangements that impacted loneliness for men. Men living in couples with children tended to be lonelier than those in childless couple households for the 35–44 and 55–64 age groups. As has been found elsewhere [47], single-parent households were also lonelier than childless couples for the 45–54 age group. In the 25–34 and 55 and older age groups, people living in various ‘other’ households, a category which included lone-person households, experienced more loneliness than those in couple households without children. Finally, loneliness was not associated with region of residence, household income, or COVID-19 lockdowns in any age group.

### Implications for practice

The findings suggest that interventions aimed at reducing loneliness should be tailored to men's specific needs at different life stages. Firstly, our findings suggest interventions should be developed to prevent men in their late 40s from experiencing loneliness, as our research suggests this group is particularly at risk. Amongst men of working age, industrial reform to increase availability of secure jobs and reduce unemployment are likely to reduce loneliness. Supporting older men (aged 65 +) to volunteer may prevent or alleviate loneliness. Public health promotion campaigns that challenge negative rhetoric around masculine gender norms may reduce loneliness amongst middle-aged men. Across age groups, preventative programs should target men who have children, who are single parents, have long-term disabilities or recently went through a breakup, and intervene early to make the most impact.

This study also highlighted policy actions that can protect men from loneliness, such as improving local neighbourhood satisfaction. Research shows ensuring that neighbourhoods have adequate and high-quality social infrastructure to support social connection can prevent loneliness [28]. Community-level interventions, that support recurrent meeting of local men in a neighbourhood, may have broad protective benefits for male loneliness [28].

Given the clear protective role of high-quality, diverse and supportive relationships across the life course, mental health and social care practitioners working with men experiencing loneliness should support them to reach out and connect to people they value to reduce loneliness. On a society-wide level, public health campaigns could promote the importance of high-quality male friendships to wellbeing, and the maintenance work required to sustaining these connections. In recognition that it is often difficult and time-intensive to change networks and that social skills interventions can have limited success in reducing loneliness, research has found that therapies that work with maladaptive social cognition (e.g., Cognitive Behavioural Therapy) can be successful in reducing loneliness [48, 49].

### Strengths and limitations

The generalisability of the current findings is limited by the Australian context, which may not correspond with the social norms and experiences of men in other cultures. Another limitation of our study is that we used a pre-existing panel dataset, which limited the measure of loneliness we could use and predictors of loneliness we could include in analyses. In future research, the use of an accepted and standardised measure of loneliness, such as the UCLA loneliness or De Jong Gierveld Loneliness scales would be preferable.

A strength of our study is that we drew on over 20 years of data, examining age-specific patterns of male loneliness. This means that we can be tentatively sure that the effects identified are age-based, rather than generational/cohort effects. However, we did not assess longitudinal trends in predictors of loneliness and consequently, causal inferences cannot be made. Future research should draw on the current findings and conduct a causational analysis of key variables.

In conclusion, this study showed that middle-aged men are at particular risk of feeling lonely. Results emphasised the need to consider social, neighbourhood and age-specific factors in understanding and addressing loneliness amongst men.

### Supplementary Information

Supplementary Material 1.

## Data Availability

This paper uses unit record data from Household, Income and Labour Dynamics in Australia Survey [HILDA] conducted by the Australian Government Department of Social Services (DSS). The findings and views reported in this paper, however, are those of the author[s] and should not be attributed to the Australian Government, DSS, or any of DSS’ contractors or partners. DOI: 10.26193/KXNEBO. The data are available upon application to the data custodian at the Australian Data Archive.
