# Supplementary material for: Predictors of male loneliness across life stages: an Australian study of longitudinal data
Source: BMC Public Health. 2024 May 10;24:1285. doi: 10.1186/s12889-024-18770-w (PMC11088127; doi:10.1186/s12889-024-18770-w)
Supplement: Supplementary file 1 — Supplementary Material 1. [file 12889_2024_18770_MOESM1_ESM.docx]

**Supplementary Information**

**Table S1: Determinants of loneliness (multi-item outcome), all age groups**

|  |  | **(1)** |  | **(2)** |
| --- | --- | --- | --- | --- |
|  |  |  |  |  |
| Age group (ref: 15-24) |  |  |  |  |
| 25-34 |  | 0.085***  (0.019) |  | 0.071***  (0.020) |
| 35-44 |  | 0.136***  (0.026) |  | 0.045*  (0.027) |
| 45-54 |  | 0.123***  (0.030) |  | -0.016  (0.034) |
| 55-64 |  | 0.038  (0.034) |  | -0.119***  (0.041) |
| 65 and older |  | -0.036  (0.037) |  | -0.208***  (0.049) |
| Labour force status (ref: Unemployed) |  |  |  |  |
| Employed |  |  |  | -0.009  (0.024) |
| Not in labour force |  |  |  | -0.040**  (0.020) |
| Job security |  |  |  | -0.030***  (0.003) |
| Log household income |  |  |  | -0.017*  (0.010) |
| Partnered |  |  |  | -0.179***  (0.021) |
| Volunteer |  |  |  | -0.007  (0.010) |
| Member of club |  |  |  | -0.023***  (0.008) |
| Life event: death of close friend |  |  |  | 0.023**  (0.010) |
| Life event: separated from spouse or partner |  |  |  | 0.200***  (0.020) |
| I seem to have many friends (1-7) |  |  |  | -0.116***  (0.004) |
| Frequency of social connection (ref: Less than once a month) |  |  |  |  |
| At least once a month |  |  |  | -0.160***  (0.012) |
| At least once a week |  |  |  | -0.313***  (0.013) |
| Household type (ref: Couple without children) |  |  |  |  |
| Couple with children |  |  |  | 0.068***  (0.013) |
| Single parent |  |  |  | 0.104***  (0.030) |
| Other household type |  |  |  | 0.156***  (0.019) |
| Region of residence (ref: Rural) |  |  |  |  |
| Major urban |  |  |  | -0.025  (0.029) |
| Other urban |  |  |  | 0.015  (0.030) |
| Satisfaction with neighbourhood (1-10) |  |  |  | -0.038***  (0.003) |
| Long-term disability |  |  |  | 0.100***  (0.012) |
| Stereotypical masculinity beliefs |  |  |  | 0.021***  (0.004) |
| COVID-19 lockdown |  |  |  | 0.051***  (0.020) |
|  |  |  |  |  |
| Mean of dependent variable |  | 2.858 |  | 2.858 |
| Observations |  | 118,394 |  | 118,394 |
| Individuals |  | 12,117 |  | 12,117 |

Note: The dependent variable is the average of a multi-item loneliness variable based on the following three statements: (i) “People don’t come to visit me as often as I would like”; (ii) “I often need help from other people but can’t get it”; and (iii) “I often feel very lonely.” The variable is the average of responses to the three statements and ranged from 1 to 7, which is increasing in the degree of reported loneliness. For job security and masculinity beliefs, indicator categories are included for missing observations and dummies are additionally included (but not shown) to control for missingness in these variables. Year dummies are included (but not shown) in all models. Standard errors are in parentheses. p < 0.01***, p < 0.05**, p < 0.10*.

**Table S2: Determinants of loneliness (multi-item outcome), by age group**

|  |  | **15-24** |  | **25-34** |  | **35-44** |  | **45-54** |  | **55-64** |  | **65+** |
| --- | --- | --- | --- | --- | --- | --- | --- | --- | --- | --- | --- | --- |
|  |  |  |  |  |  |  |  |  |  |  |  |  |
| Labour force status (ref: Unemployed) |  |  |  |  |  |  |  |  |  |  |  |  |
| Employed |  | 0.000  (0.044) |  | 0.092  (0.068) |  | -0.170**  (0.067) |  | -0.074  (0.059) |  | -0.084  (0.058) |  | -0.016  (0.122) |
| Not in labour force |  | 0.005  (0.033) |  | 0.075  (0.058) |  | -0.099  (0.066) |  | -0.066  (0.053) |  | -0.046  (0.051) |  | -0.179  (0.115) |
| Job security |  | -0.028***  (0.006) |  | -0.036***  (0.006) |  | -0.038***  (0.006) |  | -0.025***  (0.006) |  | -0.017***  (0.007) |  | -0.016  (0.013) |
| Log household income |  | 0.027  (0.027) |  | -0.075***  (0.028) |  | -0.042  (0.028) |  | -0.032  (0.025) |  | -0.009  (0.023) |  | -0.020  (0.023) |
| Partnered |  | -0.177***  (0.047) |  | -0.135***  (0.042) |  | -0.313***  (0.070) |  | -0.193***  (0.067) |  | -0.164*  (0.087) |  | -0.234***  (0.087) |
| Volunteer |  | 0.016   (0.130) |  | 0.029  (0.028) |  | 0.013  (0.024) |  | 0.008 (0.023) |  | -0.026  (0.025) |  | -0.044*  (0.025) |
| Member of club |  | -0.008  (0.021) |  | -0.018  (0.019) |  | -0.038*  (0.020) |  | -0.020  (0.022) |  | -0.021  (0.022) |  | -0.037  (0.023) |
| Life event: death of close friend |  | 0.038  (0.033) |  | -0.010  (0.033) |  | 0.030  (0.028) |  | -0.026  (0.023) |  | 0.027  (0.021) |  | 0.038**  (0.019) |
| Life event: separated from spouse or partner |  | 0.243***  (0.041) |  | 0.191***  (0.038) |  | 0.118***  (0.046) |  | 0.112**  (0.049) |  | 0.137**  (0.068) |  | 0.167**  (0.069) |
| I seem to have many friends (1-7) |  | -0.184***  (0.009) |  | -0.141***  (0.009) |  | -0.128***  (0.009) |  | -0.082***  (0.008) |  | -0.062***  (0.009) |  | -0.032***  (0.008) |
| Frequency of social connection (ref: Less than once a month) |  |  |  |  |  |  |  |  |  |  |  |  |
| At least once a month |  | -0.069  (0.045) |  | -0.213***  (0.034) |  | -0.148**  (0.027) |  | -0.150***  (0.023) |  | -0.168***  (0.027) |  | -0.119***  (0.027) |
| At least once a week |  | -0.267***  (0.043) |  | -0.363***  (0.035) |  | -0.329***  (0.030) |  | -0.260***  (0.028) |  | -0.259***  (0.029) |  | -0.221***  (0.029) |
| Household type (ref: Couple without children) |  |  |  |  |  |  |  |  |  |  |  |  |
| Couple with children |  | -0.011  (0.052) |  | 0.041  (0.026) |  | 0.079**  (0.038) |  | 0.011  (0.034) |  | 0.058*  (0.030) |  | 0.053  (0.049) |
| Single parent |  | -0.032  (0.065) |  | 0.095  (0.078) |  | 0.024  (0.097) |  | 0.225***  (0.082) |  | 0.130  (0.101) |  | 0.338***  (0.127) |
| Other household type |  | 0.012  (0.050) |  | 0.084**  (0.040) |  | 0.049  (0.069) |  | 0.155***  (0.057) |  | 0.204***  (0.060) |  | 0.313***  (0.072) |
| Region of residence (ref: Rural) |  |  |  |  |  |  |  |  |  |  |  |  |
| Major urban |  | -0.047  (0.069) |  | -0.071  (0.058) |  | 0.029  (0.077) |  | 0.027  (0.086) |  | -0.157**  (0.079) |  | 0.016  (0.010) |
| Other urban |  | 0.017  (0.073) |  | -0.035  (0.062) |  | 0.042  (0.073) |  | 0.009  (0.092) |  | -0.061  (0.080) |  | -0.019  (0.099) |
| Satisfaction with neighbourhood (1-10) |  | -0.033***  (0.006) |  | -0.035***  (0.006) |  | -0.028***  (0.007) |  | -0.034***  (0.007) |  | -0.031***  (0.008) |  | -0.025***  (0.008) |
| Long-term disability |  | 0.159***  (0.047) |  | 0.176***  (0.039) |  | 0.078**  (0.035) |  | 0.034  (0.029) |  | 0.069***  (0.026) |  | 0.078***  (0.021) |
| Stereotypical masculinity beliefs |  | 0.009  (0.011) |  | 0.016  (0.011) |  | 0.028***  (0.011) |  | 0.022**  (0.009) |  | 0.024**  (0.010) |  | 0.010  (0.008) |
| COVID-19 lockdown |  | 0.082  (0.065) |  | 0.035  (0.048) |  | -0.029  (0.050) |  | 0.027  (0.051) |  | 0.085  (0.052) |  | 0.048  (0.042) |
|  |  |  |  |  |  |  |  |  |  |  |  |  |
| Mean of dependent variable |  | 2.732 |  | 2.896 |  | 2.941 |  | 2.949 |  | 2.855 |  | 2.751 |
| Observations |  | 19,071 |  | 20,250 |  | 20,350 |  | 20,712 |  | 17,733 |  | 20,278 |
| Individuals |  | 4,072 |  | 4,433 |  | 4,042 |  | 3,783 |  | 3,202 |  | 2,674 |

Note: The dependent variable is the average of a multi-item loneliness variable based on the following three statements: (i) “People don’t come to visit me as often as I would like”; (ii) “I often need help from other people but can’t get it”; and (iii) “I often feel very lonely.” The variable is the average of responses to the three statements and ranged from 1 to 7, which is increasing in the degree of reported loneliness. For job security and masculinity beliefs, indicator categories are included for missing observations and dummies are additionally included (but not shown) to control for missingness in these variables. Year dummies are included (but not shown) in all models. Robust standard errors are in parentheses. p < 0.01***, p < 0.05**, p < 0.10*.

**Table S3: Determinants of loneliness (binary outcome), all age groups**

|  |  | **(1)** |  | **(2)** |
| --- | --- | --- | --- | --- |
|  |  |  |  |  |
| Age group (ref: 15-24) |  |  |  |  |
| 25-34 |  | 0.038  (0.048) |  | 0.100*  (0.058) |
| 35-44 |  | 0.085  (0.064) |  | 0.062  (0.086) |
| 45-54 |  | 0.150**  (0.075) |  | 0.039  (0.111) |
| 55-64 |  | 0.021  (0.084) |  | -0.143  (0.137) |
| 65 and older |  | -0.186*  (0.096) |  | -0.355**  (0.167) |
| Labour force status (ref: Unemployed) |  |  |  |  |
| Employed |  |  |  | -0.103  (0.072) |
| Not in labour force |  |  |  | -0.141**  (0.057) |
| Job security |  |  |  | -0.057***  (0.009) |
| Log household income |  |  |  | 0.010  (0.034) |
| Partnered |  |  |  | -0.813***  (0.057) |
| Volunteer |  |  |  | 0.021  (0.038) |
| Member of club |  |  |  | -0.043  (0.029) |
| Life event: death of close friend |  |  |  | 0.052  (0.034) |
| Life event: separated from spouse or partner |  |  |  | 0.431***  (0.049) |
| I seem to have many friends (1-7) |  |  |  | -0.168***  (0.008) |
| Frequency of social connection (ref: Less than once a month) |  |  |  |  |
| At least once a month |  |  |  | -0.262***  (0.033) |
| At least once a week |  |  |  | -0.453***  (0.036) |
| Household type (ref: Couple without children) |  |  |  |  |
| Couple with children |  |  |  | 0.247***  (0.043) |
| Single parent |  |  |  | 0.275***  (0.079) |
| Other household type |  |  |  | 0.442***  (0.061) |
| Region of residence (ref: Rural) |  |  |  |  |
| Major urban |  |  |  | -0.054  (0.083) |
| Other urban |  |  |  | 0.023  (0.082) |
| Satisfaction with neighbourhood (1-10) |  |  |  | -0.053***  (0.007) |
| Long-term disability |  |  |  | 0.148***  (0.034) |
| Stereotypical masculinity beliefs |  |  |  | 0.026***  (0.009) |
| COVID-19 lockdown |  |  |  | -0.084  (0.068) |
|  |  |  |  |  |
| Observations |  | 69,487 |  | 69,487 |
| Individuals |  | 6,181 |  | 6,181 |

Note: The dependent variable is the binary response to the question “I often feel very lonely”, equal to one if the response is 5-7 on the 1-7 scale, and zero otherwise. For job security and masculinity beliefs, indicator categories are included for missing observations and dummies are additionally included (but not shown) to control for missingness in these variables. Year dummies are included (but not shown) in all models. Standard errors are in parentheses. p < 0.01***, p < 0.05**, p < 0.10*.

**Table S4: Determinants of loneliness (binary outcome), by age group**

|  |  | **15-24** |  | **25-34** |  | **35-44** |  | **45-54** |  | **55-64** |  | **65+** |
| --- | --- | --- | --- | --- | --- | --- | --- | --- | --- | --- | --- | --- |
|  |  |  |  |  |  |  |  |  |  |  |  |  |
| Labour force status (ref: Unemployed) |  |  |  |  |  |  |  |  |  |  |  |  |
| Employed |  | -0.042  (0.142) |  | 0.098  (0.200) |  | -0.209  (0.209) |  | -0.255  (0.199) |  | -0.253  (0.226) |  | -0.136  (0.548) |
| Not in labour force |  | -0.113  (0.099) |  | 0.023  (0.168) |  | -0.071  (0.180) |  | -0.075  (0.182) |  | -0.061  (0.192) |  | -0.371  (0.524) |
| Job security |  | -0.047**  (0.023) |  | -0.068***  (0.023) |  | -0.082***  (0.022) |  | -0.041*  (0.022) |  | -0.038  (0.026) |  | -0.069  (0.045) |
| Log household income |  | 0.118  (0.099) |  | -0.135  (0.110) |  | 0.027  (0.106) |  | 0.032  (0.094) |  | -0.133  (0.094) |  | -0.079  (0.084) |
| Partnered |  | -1.066***  (0.175) |  | -0.967***  (0.151) |  | -0.994***  (0.213) |  | -0.870***  (0.205) |  | -0.255  (0.250) |  | -1.105***  (0.245) |
| Volunteer |  | -0.008   (0.111) |  | 0.117  (0.125) |  | 0.131  (0.104) |  | 0.033 (0.101) |  | -0.042  (0.111) |  | -0.208**  (0.097) |
| Member of club |  | -0.061  (0.076) |  | -0.146*  (0.084) |  | -0.046  (0.082) |  | -0.009  (0.082) |  | -0.023  (0.093) |  | -0.123  (0.076) |
| Life event: death of close friend |  | 0.070  (0.115) |  | 0.053  (0.120) |  | -0.044  (0.105) |  | 0.023  (0.087) |  | -0.024  (0.088) |  | 0.086  (0.064) |
| Life event: separated from spouse or partner |  | 0.661***  (0.120) |  | 0.446***  (0.116) |  | 0.288**  (0.126) |  | 0.218  (0.133) |  | 0.205  (0.171) |  | 0.190  (0.191) |
| I seem to have many friends (1-7) |  | -0.282***  (0.022) |  | -0.213***  (0.025) |  | -0.193***  (0.024) |  | -0.096***  (0.023) |  | -0.073***  (0.024) |  | -0.004  (0.020) |
| Frequency of social connection (ref: Less than once a month) |  |  |  |  |  |  |  |  |  |  |  |  |
| At least once a month |  | -0.168  (0.116) |  | -0.419***  (0.102) |  | -0.174**  (0.085) |  | -0.301***  (0.078) |  | -0.278***  (0.091) |  | -0.158*  (0.085) |
| At least once a week |  | -0.383***  (0.112) |  | -0.595***  (0.107) |  | -0.462***  (0.097) |  | -0.502***  (0.089) |  | -0.372***  (0.101) |  | -0.172*  (0.089) |
| Household type (ref: Couple without children) |  |  |  |  |  |  |  |  |  |  |  |  |
| Couple with children |  | -0.304  (0.193) |  | 0.193*  (0.114) |  | 0.307**  (0.157) |  | 0.035  (0.127) |  | 0.287**  (0.130) |  | -0.019  (0.195) |
| Single parent |  | -0.302  (0.218) |  | 0.313  (0.243) |  | 0.193  (0.298) |  | 0.285  (0.242) |  | 0.306  (0.302) |  | 0.124  (0.375) |
| Other household type |  | -0.157  (0.194) |  | 0.506***  (0.159) |  | 0.280  (0.235) |  | 0.174  (0.195) |  | 0.608***  (0.214) |  | 0.618***  (0.226) |
| Region of residence (ref: Rural) |  |  |  |  |  |  |  |  |  |  |  |  |
| Major urban |  | -0.080  (0.240) |  | -0.389*  (0.208) |  | 0.165  (0.251) |  | -0.167  (0.274) |  | -0.082  (0.330) |  | 0.330  (0.333) |
| Other urban |  | 0.190  (0.247) |  | -0.259  (0.220) |  | 0.291  (0.248) |  | -0.039  (0.276) |  | -0.170  (0.305) |  | -0.020  (0.326) |
| Satisfaction with neighbourhood (1-10) |  | -0.033**  (0.017) |  | -0.060***  (0.020) |  | -0.053***  (0.020) |  | -0.072***  (0.021) |  | -0.041*  (0.025) |  | -0.018  (0.022) |
| Long-term disability |  | 0.110  (0.123) |  | 0.384***  (0.124) |  | 0.245**  (0.110) |  | 0.004  (0.095) |  | 0.041  (0.093) |  | 0.114*  (0.065) |
| Stereotypical masculinity beliefs |  | -0.031  (0.034) |  | 0.047  (0.034) |  | 0.026  (0.035) |  | 0.017  (0.028) |  | 0.051*  (0.031) |  | 0.012  (0.020) |
| COVID-19 lockdown |  | 0.170  (0.198) |  | -0.019  (0.205) |  | -0.093  (0.219) |  | -0.113  (0.218) |  | -0.083  (0.209) |  | -0.285  (0.175) |
|  |  |  |  |  |  |  |  |  |  |  |  |  |
| Observations |  | 8,244 |  | 7,357 |  | 8,432 |  | 9,521 |  | 7,845 |  | 11,232 |
| Individuals |  | 1,427 |  | 1,278 |  | 1,336 |  | 1,423 |  | 1,175 |  | 1,230 |

Note: The dependent variable is the binary response to the question “I often feel very lonely”, equal to one if the response is 5-7 on the 1-7 scale, and zero otherwise. For job security and masculinity beliefs, indicator categories are included for missing observations and dummies are additionally included (but not shown) to control for missingness in these variables. Year dummies are included (but not shown) in all models. Robust standard errors are in parentheses. p < 0.01***, p < 0.05**, p < 0.10*.
